# Supplementary material for: Risk factors for hypotony after transconjunctival sutureless vitrectomy
Source: PLoS One. 2025 Apr 28;20(4):e0321135. doi: 10.1371/journal.pone.0321135 (PMC12036930; doi:10.1371/journal.pone.0321135)
Supplement: S1 Table — (DOCX) [file pone.0321135.s001.docx]

**S1 Table** Demographics of patients without tamponade and the analysis findings for risk factors in the postoperative hypotony and non-hypotony groups

|  | **Hypotony**  **(n = 90)** | **Non-hypotony**  **(n = 349)** | **Logistic Regression** | | | | | |
| --- | --- | --- | --- | --- | --- | --- | --- | --- |
|  |  |  | **Univariate Analysis** | | | **Multivariate Analysis** | | |
|  |  |  | **OR** | **95% CI** | ***P* value** | **OR** | **95% CI** | ***P* value** |
| **Age, years** | 61.7 ± 12.1 | 64.5 ± 13.2 | 1.02 | 1.00–1.03 | 0.073 | 1.01 | 0.99–1.03 | 0.240 |
| **Sex** |  | | | | | | | |
| Male | 45 (50.0) | 199 (57.0) | 0.75 | 0.47–1.20 | 0.232 |  |  |  |
| Female | 45 (50.0) | 150 (43.0) |  |  |  |  |  |  |
| **Laterality** |  | | | | | | | |
| Right eye | 48 (53.3) | 169 (48.4) | 1.22 | 0.77–1.94 | 0.406 |  | | |
| Left eye | 42 (46.7) | 180 (51.6) |  | | | | | |
| **Preoperative IOP, mmHg** | 15.0 ± 5.6 | 14.3 ± 5.8 | 0.98 | 0.95–1.02 | 0.307 |  |  |  |
| **Axial length, mm** | 25.0 ± 2.2 | 24.3 ± 1.6 | 0.82 | 0.72–0.92 | 0.005** | 0.83 | 0.73–0.94 | 0.003** |
| **Gauge number** |  | | | | | | | |
| 25 G | 72 (80.0) | 290 (83.1) | 0.81 | 0.45–1.46 | 0.491 |  | | |
| 27 G | 18 (20.0) | 59 (16.9) |  | | | | | |
| **Previous vitrectomy** |  | | | | | | | |
| Yes (≥2 times) | 16 (17.8) | 35 (10.0) | 1.94 | 1.02–3.69 | 0.041* | 1.23 | 0.54–2.80 | 0.630 |
| No (1 time) | 74 (82.2) | 314 (90.0) |  | | | | | |
| **Silicone oil removal** |  | | | | | | | |
| Yes | 8 (8.9) | 10 (2.9) | 3.31 | 1.27–8.64 | 0.017* | 2.43 | 0.73–8.10 | 0.147 |
| No | 82 (91.1) | 339 (97.1) |  | | | | | |
| **TA usage** |  | | | | | | | |
| Yes | 75 (83.3) | 312 (89.4) | 0.59 | 0.31–1.14 | 0.112 |  | | |
| No | 15 (16.7) | 37 (10.6) |  | | | | | |
| **Crystalline lens bag status** |  | | | | | | | |
| One chamber | 12 (13.3) | 60 (17.2) | 0.74 | 038–1.45 | 0.378 |  | | |
| Two chambers | 78 (86.7) | 289 (82.8) |  | | | | | |
| **Vitrectomy experience** |  | | | | | | | |
| >4 years | 71 (78.9) | 270 (77.4) | 1.09 | 0.62–1.92 | 0.757 |  | | |
| ≤4 years | 19 (21.1) | 79 (22.6) |  | | | | | |
| **Surgical time, min** | 44.1 ± 20.1 | 44.6 ± 23.0 | 1.00 | 0.99–1.01 | 0.860 |  | | |

**P* < 0.05, ***P* < 0.01

Note*:* Data are expressed as the number (%) or as the mean ± standard deviation. Percentages represent the proportion of patients in the hypotony and non-hypotony groups.

OR, odds ratio; CI, confidence interval; IOP, intraocular pressure; TA, triamcinolone acetonide
